# Awareness, attitudes, and self-reported clinical management of antimicrobial-associated neurocognitive adverse effects among healthcare providers in Saudi Arabia

**DOI:** 10.3389/fphar.2026.1866022

**Published:** 2026-06-18

**Authors:** Kousalya Prabahar, Wejdan Alghamdi, Hind Alshehri, Shatha Alharbi, Hanan Alshareef, Yasser Alatawi, Sawsan Zaitone, Ahmed Mohsen Elsaid Hamdan

**Affiliations:** 1 Department of Pharmacy Practice, Faculty of Pharmacy, University of Tabuk, Tabuk, Saudi Arabia; 2 PharmD Program, Faculty of Pharmacy, University of Tabuk, Tabuk, Saudi Arabia; 3 Department of Pharmacology and Toxicology, Faculty of Pharmacy, University of Tabuk, Tabuk, Saudi Arabia

**Keywords:** antimicrobial stewardship, antimicrobials, awareness, neurocognitive adverse effects, pharmacovigilance, Saudi Arabia

## Abstract

**Background:**

Antimicrobials are among the most frequently prescribed medications worldwide, yet their neurocognitive adverse effects remain underrecognized. The adverse effects range from mild symptoms to serious effects and unrecognized adverse effects may influence prescribing behaviors and inappropriate continuation or discontinuation of antimicrobial therapy. Evidence on healthcare providers’ awareness, attitudes, and self-reported clinical management regarding neurocognitive risks is limited, especially in Saudi Arabia. This study aimed to evaluate healthcare providers’ awareness, attitudes, and self-reported clinical management of antimicrobial-associated neurocognitive adverse effects in Saudi Arabia.

**Methods:**

A cross-sectional survey was conducted among healthcare providers in primary, secondary, and tertiary healthcare settings. A structured, self-administered questionnaire assessed awareness, attitudes, and self-reported clinical management related to neurocognitive adverse effects. Descriptive statistics and inferential analyses (Chi-square/Fisher’s exact tests) were performed.

**Results:**

Among 370 participants, 41.1% reported encountering neurological or cognitive symptoms during antimicrobial therapy, most commonly confusion and agitation. Fluoroquinolones were most frequently implicated (61.9%). High awareness was observed in 31.1%, while 27.0% demonstrated low awareness. Positive attitudes toward considering neurocognitive risks were reported by 74.6%. Most providers (94.1%) reported appropriate clinical management of suspected neurocognitive adverse effects, but only 46.2% reported consistent documentation. Physicians demonstrated significantly higher awareness, more positive attitudes, and more appropriate management than other professions (p < 0.05).

**Conclusion:**

Important gaps in awareness and documentation of neurocognitive adverse effects persist among healthcare providers. Strengthening multidisciplinary education, integrating neurocognitive safety into antimicrobial stewardship programs, and improving reporting systems are essential to enhance patient safety.

## Introduction

Antimicrobials remain among the most frequently prescribed therapeutic agents worldwide and have profoundly reduced morbidity and mortality associated with infectious diseases (Olaru et al., 2025). However, inappropriate antimicrobial use continues to undermine these benefits, contributing to antimicrobial resistance, avoidable adverse effects, suboptimal patient outcomes, and increased healthcare burden (Kousalya et al., 2010; Oliveira et al., 2024). While efforts to optimize antimicrobial use have primarily focused on resistance containment and treatment outcomes (Capuozzo et al., 2024), comparatively less attention has been paid to their safety profile, particularly neurocognitive adverse effects.

Antimicrobial-associated neurocognitive adverse effects include a broad spectrum of clinical presentations, ranging from mild symptoms such as insomnia, to severe conditions including delirium, and seizures (Althubyani et al., 2024). These effects are particularly relevant in middle-aged and older adults, who are more vulnerable due to age-related physiological changes, multimorbidity, renal or hepatic dysfunction, and polypharmacy (Soraci et al., 2023; Prabahar et al., 2024; Prabahar, 2025). In clinical practice, these adverse effects are often underrecognized, potentially resulting in inappropriate continuation or escalation of antimicrobial therapy.

Several biological mechanisms have been proposed to explain antimicrobial-induced neurocognitive adverse effects. Disruption of the gut–brain axis may influence neuroinflammatory pathways and neurotransmitter signaling, thereby affecting cognition and behavior (Diamanti et al., 2022; Kandpal et al., 2022; You et al., 2024; Sołek et al., 2025). In addition, certain antimicrobial classes exert direct effects on the central nervous system. For example, β-lactams and fluoroquinolones can antagonize gamma-aminobutyric acid (GABA) receptors, leading to enhanced neuronal excitability and a higher risk of seizures and altered mental status (Radkowski et al., 2025). Other agents, such as metronidazole were associated with reversible encephalopathy and peripheral neuropathy (Goolsby et al., 2018). These mechanisms underscore the importance of recognizing antimicrobial-associated neurocognitive risks in clinical decision-making.

Globally, awareness of antimicrobial-related neurocognitive adverse effects is increasing; however, studies evaluating healthcare providers’ awareness, attitudes, and clinical practices remain limited. Several studies have been conducted in Saudi Arabia assessing antimicrobial-related knowledge, attitudes, and prescribing practices among different populations. For example, Alkhuraisi et al. evaluated public knowledge and attitudes toward antibiotic resistance in Saudi Arabia (Alnasser et al., 2021; Alkhuraisi et al., 2023), reporting variable awareness levels among the general population. Similarly, other cross-sectional studies have examined physicians’ prescribing behaviors and healthcare workers’ understanding of antimicrobial use, resistance, and stewardship in clinical settings (Abdallah et al., 2025; Hariri et al., 2026). However, these studies primarily focused on antimicrobial resistance, prescribing patterns, and general antibiotic awareness and there is a lack of research specifically addressing antimicrobial-associated neurocognitive adverse effects, particularly among healthcare professionals.

Healthcare delivery involves a multidisciplinary team, including physicians, pharmacists, and nurses, all of whom play important roles in antimicrobial prescribing, monitoring, and adverse event recognition (Alshahrani et al., 2026). Evaluating awareness, attitudes, and clinical management practices across these professional groups can provide valuable insights into gaps in knowledge and practice that may impact patient safety.

Therefore, this study aims to assess healthcare providers’ awareness, attitudes, and self-reported clinical management of antimicrobial-associated neurocognitive adverse effects in Saudi Arabia.

## Methods

### Study design and setting

A cross-sectional study was conducted to assess healthcare providers’ awareness, attitudes, and self-reported clinical management of antimicrobial-associated neurocognitive adverse effects. The study was carried out across healthcare settings in Saudi Arabia, including primary healthcare centers, and secondary and tertiary hospitals in both governmental and private sectors.

### Study population

The study population included healthcare providers involved in antimicrobial-related patient care, including physicians, pharmacists, nurses, and allied health professionals.

### Inclusion and exclusion criteria

Participants were eligible if they:

- Were currently employed in healthcare settings in Saudi Arabia
- Were involved in antimicrobial-related clinical activities
- Provided informed consent

Participants were excluded if they:

- Had no involvement in antimicrobial use or patient care
- Submitted incomplete questionnaires

### Sample size determination

The sample size was calculated using G*Power software with a confidence level of 95% and a margin of error of ±5%. Based on national estimates of approximately 376,891 healthcare providers, the minimum required sample size was 358. To account for potential incomplete responses, a target of 370 participants was set.

### Sampling technique and recruitment

A non-probability snowball sampling technique was used. Initial participants were recruited through professional networks and institutional contacts and were asked to share the survey with eligible colleagues. Participants were invited to complete the questionnaire via a secure, digitally distributed Google Forms link. Due to the open distribution and snowball recruitment approach, the total number of individuals who received the survey invitation could not be determined; therefore, a response rate could not be calculated, and non-response bias could not be formally assessed.

### Data collection instrument

The questionnaire was developed based on an extensive literature review (Appa et al., 2017; Payne et al., 2017; Wierzbiński et al., 2023; Althubyani et al., 2024; Anwar et al., 2024; Xie et al., 2024). Content validity was evaluated by five experts in infectious diseases and antimicrobial stewardship. A pilot test involving 15 healthcare providers was conducted to assess clarity, and minor revisions were made.

The final questionnaire included five sections:

➢ Demographic and professional characteristics
➢ Antimicrobial exposure and clinical practice patterns
➢ Awareness of neurocognitive adverse effects
➢ Attitudes toward neurocognitive risk
➢ Self-reported clinical management

### Awareness assessment

Awareness was assessed using ten multiple-choice questions. Each correct answer was given 1 point and the awareness scores were calculated based on the number of correct responses. Scores were categorized as high (8–10), moderate (5–7), and low (0–4). The internal consistency of the awareness scale was acceptable (Cronbach’s α = 0.72).

### Attitude assessment

Attitudes toward antimicrobial prescribing and its potential neurological and cognitive adverse effects were assessed using six statements rated on a five-point Likert scale (Never, Rarely, Sometimes, Often, Always). Responses were scored from 1 to 5, with higher scores indicating a more positive and proactive attitude toward safe antimicrobial prescribing and neurocognitive risk awareness. The total attitude score ranged from 6 to 30. Attitude levels were categorized as negative (6–17) and positive (18–30), using a midpoint-based cut-off corresponding to 60% of the maximum score. The attitude scale demonstrated good internal consistency (Cronbach’s α = 0.81).

### Self-reported clinical management

Clinical management was assessed using a scenario-based question asking participants how they would respond when neurological symptoms appear in a patient receiving antimicrobial therapy. Additional items evaluated documentation and reporting practices. Responses were classified as appropriate when they reflected recognized management strategies for suspected antimicrobial-associated neurocognitive adverse effects, including specialist consultation, discontinuation when clinically justified, or switching to an alternative antimicrobial when the suspected neurotoxicity outweighed the therapeutic benefit and a suitable effective alternative was available.

### Statistical analysis

Data were analyzed using SPSS version 27. Descriptive statistics summarized participant characteristics. Continuous variables were reported as medians (IQR), and categorical variables as frequencies and percentages. Associations were assessed using Chi-square or Fisher’s exact tests. A p-value of <0.05 was considered statistically significant.

### Ethical considerations

The study was approved by the Local Research Ethics Committee of the University of Tabuk (Approval No. UT-692-316-2025) and conducted in accordance with the Declaration of Helsinki. Participation was voluntary, and electronic informed consent was obtained through a mandatory consent statement prior to accessing the questionnaire. All responses were anonymized, and no identifiable information was collected.

## Results

### Participant characteristics

A total of 370 healthcare providers participated in the study. The majority were female (60%), with a median age of 44 years (IQR: 38–51). Physicians constituted the largest professional group (41.9%), followed by pharmacists (33.0%). More than half of participants (52.4%) reported over 10 years of clinical experience. Participants were distributed across healthcare settings (Table 1).

**TABLE 1:** Demographic and professional characteristics of participants.

Variable	n (%)
Gender Female Male	222 (60.0) 148 (40.0)
Age (years) Median (IQR)	44 (38–51)
Profession Physicians Pharmacists Nurses Others	155 (41.9) 122 (33.0) 81 (21.9) 12 (3.2)
Years of experience Median (IQR)	11 (6–17)
Practice type Governmental hospital Private hospital Primary healthcare center	185 (50.0) 111 (30.0) 74 (20.0)

### Antimicrobial exposure and clinical experience

Nearly half of participants (47.0%, n = 174) reported regular involvement in antimicrobial prescribing, dispensing, or administration four or more times per month. The most frequently reported antimicrobials encountered in clinical practice were ceftriaxone (27.8%), amoxicillin (21.9%), azithromycin (14.0%), and meropenem (11.0%). Overall, 41.1% (n = 152) of participants reported encountering neurocognitive adverse effects in patients during antimicrobial therapy.

### Neurocognitive adverse effects

**FIGURE 1:**
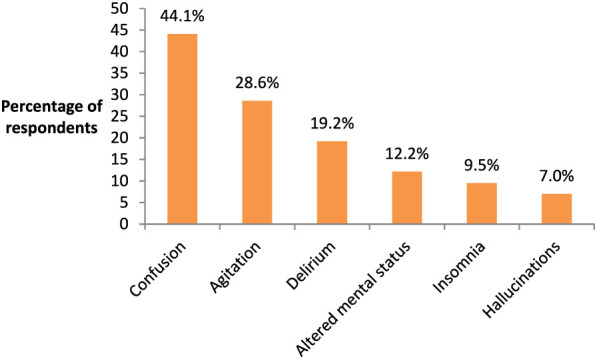
Neurocognitive adverse effects during antimicrobial therapy.

The most frequently reported neurocognitive adverse effect was confusion, followed by agitation (Figure 1).

### Antimicrobial classes associated with neurocognitive adverse effects

Fluoroquinolones were the most commonly reported antimicrobial class associated with neurocognitive adverse effects (61.9%), followed by macrolides (38.1%), β-lactams (28.9%), and carbapenems (17.0%).

### Awareness of antimicrobial-associated neurocognitive adverse effects

**FIGURE 2:**
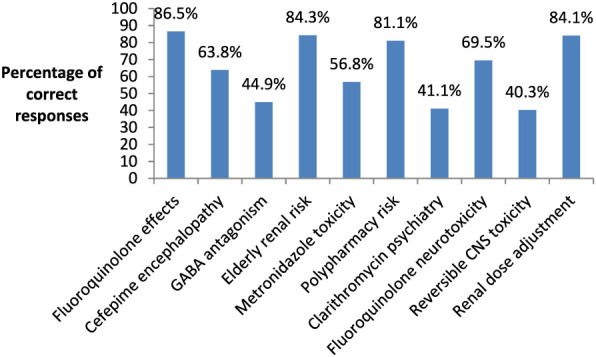
Awareness levels of antimicrobial-associated neurocognitive adverse effects.

Based on the 10-item awareness assessment, 31.1% of participants exhibited high awareness, while 41.9% and 27.0% demonstrated moderate and low awareness. Correct response rates varied across the items, with the highest awareness observed for fluoroquinolone-associated neurotoxicity (86.5%) and patient-related risk factors (84.3%) (Figure 2).

### Attitudes Toward Neurocognitive Risk

**FIGURE 3:**
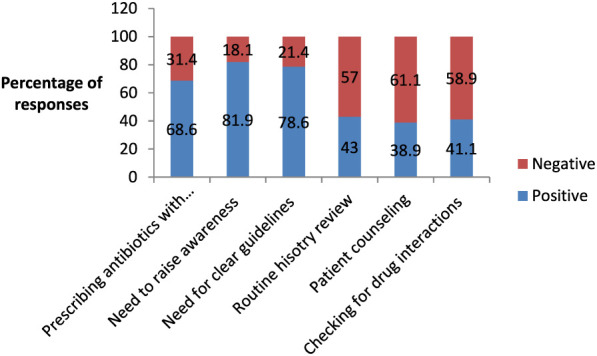
Attitudes toward neurocognitive risk and antimicrobial safety.

Overall, 74.6% of healthcare providers demonstrated a positive attitude toward antimicrobial prescribing with consideration of neurocognitive adverse effects, while 25.4% showed a negative attitude. The highest levels of agreement were observed for the need to raise awareness (81.9%, n = 303) and support for clear prescribing guidelines (78.6%, n = 291). In contrast, consistent patient counseling regarding potential neurological side effects was less frequently reported (38.9%, n = 144) (Figure 3).

### Self-reported clinical management

**FIGURE 4:**
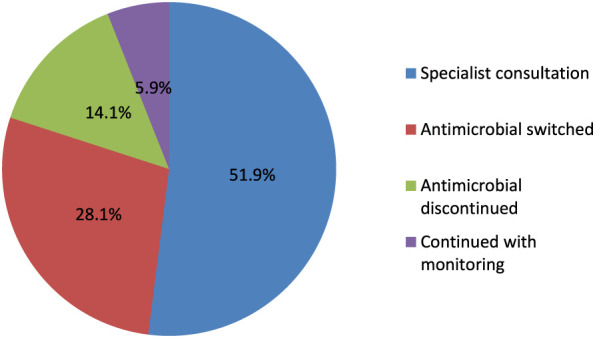
Clinical actions taken when neurocognitive symptoms occur.

Figure 4 shows the clinical management of healthcare professionals regarding the management of neurocognitive symptoms during antimicrobial therapy. Most participants demonstrated appropriate practice, with 348 (94.1%) reporting adherence to recommended procedures, while 22 (5.9%) exhibited inappropriate practice. When such symptoms were suspected, the most commonly reported actions were consultation with a specialist (51.9%) and switching to an alternative antimicrobial (28.1%). However, only 46.2% of participants reported consistent documentation of suspected neurocognitive adverse effects in medical records or reporting systems.

### Association between demographics and study outcomes

No statistically significant associations were observed between awareness, attitudes, or clinical management and gender, age group, or healthcare setting (p > 0.05). In contrast, profession demonstrated a statistically significant association with all three domains. Physicians exhibited higher awareness, more positive attitudes, and more appropriate clinical management compared with pharmacists, nurses, and other healthcare professionals (p < 0.05). Years of experience was significantly associated with awareness only (p = 0.03), with more experienced participants showing higher awareness levels (Table 2).

**TABLE 2:** Association between demographics and study outcomes.

Variable	High awareness n (%)	Positive attitude n (%)	Appropriate management n (%)	p-value (Awareness)	p-value (Attitude)	p-value (Management)
Gender Female Male	67 (30.2) 47 (31.8)	182 (82.0) 116 (78.4)	210 (94.6) 138 (93.2)	0.45	0.47	0.62
Age (years) <30 31–40 41–50 >50	6 (13.0) 27 (29.3) 42 (32.1) 19 (18.8)	32 (69.6) 67 (72.8) 102 (77.9) 83 (82.2)	42 (91.3) 88 (95.7) 123 (93.9) 91 (90.1)	0.52	0.50	0.54
Profession Physicians Pharmacists Nurses Others	60 (38.7) 32 (26.2) 16 (19.8) 4 (33.3)	137 (88.4) 94 (77.0) 57 (70.4) 10 (83.3)	150 (96.8) 114 (93.4) 73 (90.1) 11 (91.7)	0.02*	0.02*	0.03*
Years of experience <5 5–10 >10	15 (21.4) 31 (29.2) 68 (35.1)	50 (71.4) 81 (76.4) 167 (86.1)	64 (91.4) 101 (95.3) 183 (94.3)	0.03*	0.37	0.21
Practice type Governmental hospital Private hospital Primary healthcare center	47 (25.4) 39 (35.1) 28 (37.8)	157 (84.9) 89 (80.2) 52 (70.3)	176 (95.1) 102 (91.9) 70 (94.6)	0.29	0.41	0.44

* Statistically significant.

## Discussion

This multicenter study provides valuable insights into healthcare providers’ awareness, attitudes, and self-reported management of antimicrobial-associated neurocognitive adverse effects in Saudi Arabia. While awareness and attitudes were generally favorable, important gaps remain, notably in documentation practices and interprofessional variability.

Only one-third of participants demonstrated high awareness, which aligns with existing literature suggesting that antimicrobial safety, particularly neurotoxicity, is less emphasized than resistance and efficacy (Grill and Maganti, 2011; Mattappalil and Mergenhagen, 2014; Warstler and Bean, 2016; Moynan et al., 2022; Althubyani et al., 2024). The relatively high recognition of fluoroquinolone-associated neurotoxicity in this study likely reflects increasing global regulatory warnings and clinical attention to this class (Shamim et al., 2026). Although recognition of fluoroquinolone-associated neurotoxicity was high, awareness of other antimicrobial classes was comparatively limited, suggesting uneven awareness distribution.

Encouragingly, attitudes toward antimicrobial safety were largely positive, with most participants acknowledging the importance of considering neurocognitive adverse effects and the need for clearer clinical guidelines. This finding is critical, as attitudes often serve as a precursor to behavioral change in clinical practice (Glasman and Albarracín, 2006). Nonetheless, the discrepancy between positive attitudes and suboptimal patient counseling rates highlights a well-documented awareness-clinical management practice gap. Time constraints, competing clinical priorities, and uncertainty about risk communication may contribute to this gap (Parveen et al., 2025; Shalali et al., 2025). Addressing these barriers through structured antimicrobial stewardship programs and practical clinical tools may facilitate translation of awareness into consistent practice.

Although most participants reported appropriate clinical management, this finding should be interpreted cautiously due to the reliance on self-reported responses. The relatively high proportion of participants reporting appropriate clinical management despite lower levels of high awareness may reflect the influence of general clinical protocols, multidisciplinary consultation, and routine antimicrobial stewardship practices rather than detailed awareness of antimicrobial-associated neurocognitive adverse effects specifically. It is also possible that some participants overestimated their clinical practices due to social desirability bias, a recognized limitation of self-reported survey studies. Participants may have selected responses perceived as professionally appropriate even if actual practice varies in routine clinical settings. Most respondents indicated that they would modify or discontinue the suspected antimicrobial and/or seek specialist consultation, which is consistent with recommended management strategies for drug-induced neurotoxicity (Grill and Maganti, 2011; Zhang et al., 2024). However, modification or switching of antimicrobial therapy should be individualized and guided by antimicrobial stewardship principles. Unnecessary discontinuation or replacement of an optimal antimicrobial may contribute to treatment failure, broader-spectrum antimicrobial exposure, or antimicrobial resistance. Therefore, management decisions should incorporate assessment of infection severity, microbiological data, patient-specific risk factors, and the likelihood that the antimicrobial is responsible for the neurological symptoms. However, fewer than half only consistently documented adverse events, representing a critical gap in pharmacovigilance. Poor documentation undermines continuity of care and limits adverse event reporting systems (Demsash et al., 2023). Although our study did not directly assess reasons for underreporting or poor documentation, several factors may contribute to this finding. These may include limited awareness of pharmacovigilance reporting systems, time constraints in busy clinical settings, uncertainty regarding causality, and underestimation of the clinical significance of neurocognitive adverse effects, particularly when symptoms are mild or transient. Integration of simplified electronic documentation and reporting prompts within antimicrobial stewardship or electronic medical record systems may represent the most practical strategy to improve reporting consistency. Such systems could facilitate timely recognition, standardized documentation, and pharmacovigilance reporting while minimizing additional workload for healthcare providers.

Physicians demonstrated higher awareness and management compared with other professionals, highlighting disparities within multidisciplinary teams. Given the key role of pharmacists and nurses in monitoring and patient interaction (Ravi et al., 2022), targeted interprofessional education is essential (Alharthi et al., 2021).

The association between years of experience and awareness further supports the role of cumulative clinical exposure in recognizing adverse drug effects. However, reliance on experiential learning alone is insufficient, particularly in rapidly evolving areas such as antimicrobial safety. Structured continuing professional development and incorporation of neurotoxicity content into clinical guidelines may help bridge this gap, particularly for less experienced practitioners (Ali et al., 2025; Yaroslavtseva et al., 2025).

## Limitations

The use of snowball sampling technique introduces potential selection bias and limits generalizability, as participants were recruited through professional networks and may not be fully representative of all healthcare providers in Saudi Arabia. Furthermore, because the survey was distributed using an open snowball sampling approach, the total number of healthcare providers who received the survey invitation was unknown. Consequently, the response rate could not be calculated, and the potential for non-response bias could not be formally evaluated. Participants with greater interest or awareness regarding antimicrobial safety may have been more likely to participate, potentially influencing the observed awareness and attitude estimates. The distribution of participants across professions was uneven, with physicians comprising the largest proportion of respondents. This may have led to the underrepresentation of other key professional groups, potentially limiting the generalizability of interprofessional comparisons. The reliance on self-reported data may introduce response bias, including social desirability bias, whereby participants may overreport appropriate clinical practices or awareness levels. In particular, the discrepancy between moderate awareness levels and high reported appropriate management may partly reflect overestimation of actual clinical behavior. The study did not include objective validation methods, such as medical record review, pharmacovigilance audits, or direct observation of practice, to verify whether self-reported management accurately reflected real-world clinical behavior. Future studies incorporating objective practice assessment are warranted.

## Conclusion

While healthcare providers in Saudi Arabia demonstrated generally positive attitudes toward antimicrobial safety and appropriate self-reported management of suspected neurocognitive adverse effects, important gaps persist, particularly in awareness levels and documentation practices. Only a minority of participants demonstrated high awareness, and less than half consistently documented suspected adverse effects. These findings highlight the need to strengthen antimicrobial stewardship programs, improve interprofessional education, and enhance pharmacovigilance systems to ensure more consistent recognition and reporting of antimicrobial-associated neurocognitive adverse effects.

## Data Availability

The original contributions presented in the study are included in the article/supplementary material, further inquiries can be directed to the corresponding author.
